# Meta-Analysis of Usefulness of Cerebral Embolic Protection During Transcatheter Aortic Valve Implantation

**DOI:** 10.1016/j.amjcard.2021.01.023

**Published:** 2021-05-01

**Authors:** Yousif Ahmad, James P. Howard

**Affiliations:** a Smidt Heart Institute, Cedars-Sinai Medical Center, Los Angeles, California; b National Heart and Lung Institute, Imperial College London, London, United Kingdom

## Abstract

One of the most feared complications of transcatheter aortic valve implantation (TAVI) is stroke, with increased mortality and disability observed in patients suffering a stroke after TAVI. There has been no significant decline in stroke rates seen over the last 5 years; attention has therefore been given to strategies for cerebral embolic protection. With the emergence of new randomized trial data, we sought to perform an updated systematic review and meta-analysis to examine the effect of cerebral embolic protection during TAVI both on clinical outcomes and on neuroimaging parameters. We performed a random-effects meta-analysis of randomized clinical trials of cerebral embolic protection during TAVI. The primary end point was the risk of stroke. The risk of stroke was not significantly different with the use of cerebral embolic protection: relative risk (RR) 0.88, 95% confidence interval (CI) 0.57 to 1.36, p = 0.566. Nor was there a significant reduction in the risk of disabling stroke, non-disabling stroke or death. There was no significant difference in total lesion volume on MRI with cerebral embolic protection: mean difference -74.94, 95% CI -174.31 to 24.4, p = 0.139. There was also not a significant difference in the number of new ischemic lesions on MRI: mean difference -2.15, 95% -5.25 to 0.96, p = 0.176, although there was significant heterogeneity for the neuroimaging outcomes. In conclusion, cerebral embolic protection during TAVI is safe but there is no evidence of a statistically significant benefit on clinical outcomes or neuroimaging parameters.

The clinical trial evidence base for transcatheter aortic valve implantation (TAVI) is as compared with surgical aortic valve replacement (SAVR) has now been established across the spectrum of surgical risk.^1^, ^2^, ^3^, ^4^, ^5^, ^6^ One of the most feared complications of TAVI is stroke, with increased mortality and disability observed in patients suffering a stroke after TAVI.^7^ Furthermore, it is not known whether silent cerebral lesions may lead to deterioration in neurocognitive function in the longer-term. Despite advances in TAVI technology and technique, in-hospital stroke rates are still in the order of 2%. Attention has therefore been given to strategies for cerebral embolic protection, where devices can be employed to either filter or deflect debris during TAVI. Embolic protection devices have thus far been tested in relatively small randomized clinical trials (RCTs) only; with the emergence of new randomized trial data, we sought to perform an updated systematic review and meta-analysis to examine the effect of cerebral embolic protection during TAVI both on clinical outcomes and on neuroimaging parameters.

## Methods

The present analysis was performed according to published PRISMA guidance.^8^ We prospectively registered the analysis at the PROSPERO international prospective register of systematic reviews (CRD42020214106). Ethical approval was not applicable in this case.

We performed a systematic search of the MEDLINE, Cochrane Central Register of Controlled Trials, and Embase databases from December 2010 through October 2020 for all randomized trials comparing cerebral embolic protection to control during TAVI. Our search strings included (“severe aortic stenosis” OR “severe symptomatic aortic stenosis”) AND (“transcatheter aortic valve implantation” OR “transcatheter aortic valve replacement”) AND (“embolic protection”) OR (“cerebral protection”). We hand-searched the bibliographies of selected studies and meta-analyses to identify further eligible studies. There were no language restrictions. We also reviewed abstracts presented at conferences. Abstracts were reviewed for suitability and articles accordingly retrieved. Two independent authors performed the search and literature screening (YA and JH), with disputes resolved by consensus.

Only RCTs comparing cerebral embolic protection to control were included. We did not consider observational studies

The primary end point was the risk of stroke. Other clinical end points included risk of death, disabling stroke, non-disabling stroke, all bleeding, life-threatening or disabling bleeding, major vascular complications, and acute kidney injury. Neuroimaging endpoints were total lesion volume on MRI, new ischemic lesions on MRI, and the number of patients with new ischemic lesions on MRI.

Two authors (YA and JH) independently abstracted the data from included trials, with disputes resolved by consensus. Tests for publication bias would only be performed in the event of 10 or more trials being suitable for inclusion.^9^ Included studies were assessed using the Cochrane Risk of Bias tool.^10^

Intention-to-treat analyses were used, with the longest follow-up time available. For clinical outcomes, we extracted event counts to calculate relative risks (RR) and performed random-effects meta-analyses using the restricted maximum likelihood estimator, with fixed effect as a sensitivity analysis. For imaging end points, we performed a random-effects meta-analysis using the mean difference in effect sizes and their associated standard errors using the restricted maximum likelihood (REML) estimator. The standard errors for the trials were calculated by dividing the difference between the upper and lower 95% confidence intervals by 2 × the appropriate normal score (1.96). Interactions between important characteristics that varied across trials were assessed by performing a mixed-effects meta-analysis with the characteristic as a moderator. We performed the same analyses with standardized mean differences as a sensitivity analysis. Medians and interquartile ranges were converted to means and standard errors using published methodology.^11^

The I^2^ statistic was used to assess heterogeneity.^12^ Low heterogeneity was defined as 0-25%; moderate heterogeneity was defined as 25% to 50%; and significant heterogeneity was defined as >50%. Mean values are expressed as mean ± SD unless otherwise stated. Statistical significance was set at p <0.05. The statistical programming environment R^13^ with the metafor package^13^ was used for all statistical analyses.

## Results

Six trials^14^, ^15^, ^16^, ^17^, ^18^, ^19^ randomizing 856 patients were eligible for analysis. 488 patients were randomized to cerebral embolic protection and 368 patients were randomized to control. Baseline characteristics are shown in Table 1 of the Supplementary Appendix. The risk of bias assessment is shown in Table 2 of the Supplementary Appendix. The search strategy and results are shown in Figure 1 of the Supplementary Appendix.

**Figure 1:**
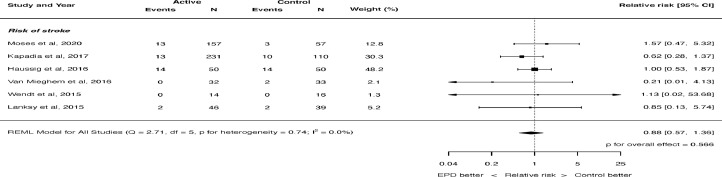
Effect of cerebral embolic protection on the risk of all stroke. REML = restricted maximum likelihood. Q = Cochran's Q level of heterogeneity; df = degrees of freedom; EPD = embolic protection device.

**Figure 2:**
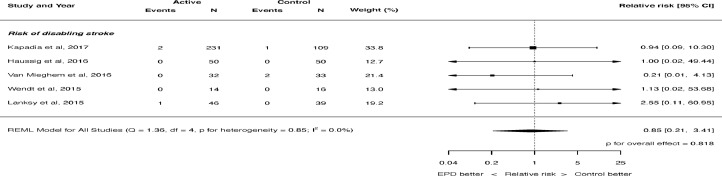
Effect of cerebral embolic protection on the risk of disabling stroke. REML = restricted maximum likelihood.

**Figure 3:**
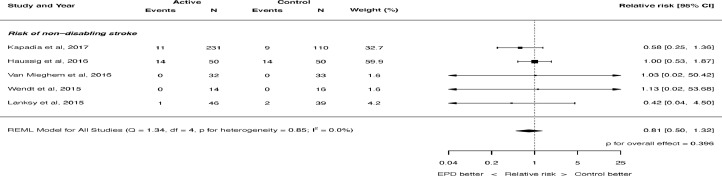
Effect of cerebral embolic protection on the risk of non-disabling stroke. REML = restricted maximum likelihood.

The risk of stroke was not significantly different with the use of cerebral embolic protection: RR 0.88, 95% CI 0.57 to 1.36, p = 0.566 (see Figure 1). There was no heterogeneity (I^2^ = 0.0%). There was also no significant difference in the risk of disabling stroke (RR 0.85, 95% CI 0.21 to 3.41, p = 0.818, Figure 2) or non-disabling stroke (RR 0.81, 95% CI 0.50 to 1.32, p = 0.396, Figure 3). Again, there was no heterogeneity for either of these outcomes (I^2^ = 0.0%). The risk of death was not significantly different with the use of cerebral embolic protection: RR 0.56, 95% CI 0.21 to 1.51, p = 0.255. There was no heterogeneity (I^2^ = 0.0%).

The risk of all bleeding was not significantly different with the use of cerebral embolic protection (RR 0.84, 95% CI 0.55 to 1.29, p = 0.431). There was mild heterogeneity (I^2^=13.5%). The risk of life-threatening or disabling bleeding was also not significantly different with the use of cerebral embolic protection (RR 0.71, 95% CI 0.21 to 2.40, p = 0.587). There was mild heterogeneity (I^2^=14.4%). The risk of major vascular complications was not significantly different with the use of cerebral embolic protection: RR 1.04, 95% 0.62 to 1.74, p = 0.877. There was no heterogeneity (I^2^ = 0.0%). Nor was there any difference in the risk of acute kidney injury: RR 0.75, 95% CI 0.21 to 2.76, p = 0.668. There was mild heterogeneity (I^2^ = 6.2%).

**Figure 4:**
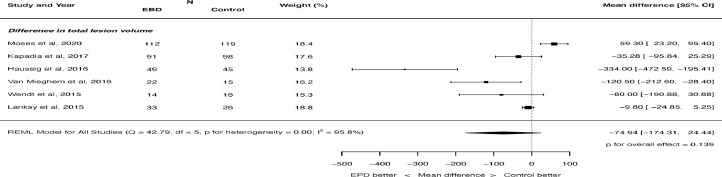
Effect of cerebral embolic protection on total lesion volume. REML = restricted maximum likelihood.

**Figure 5:**
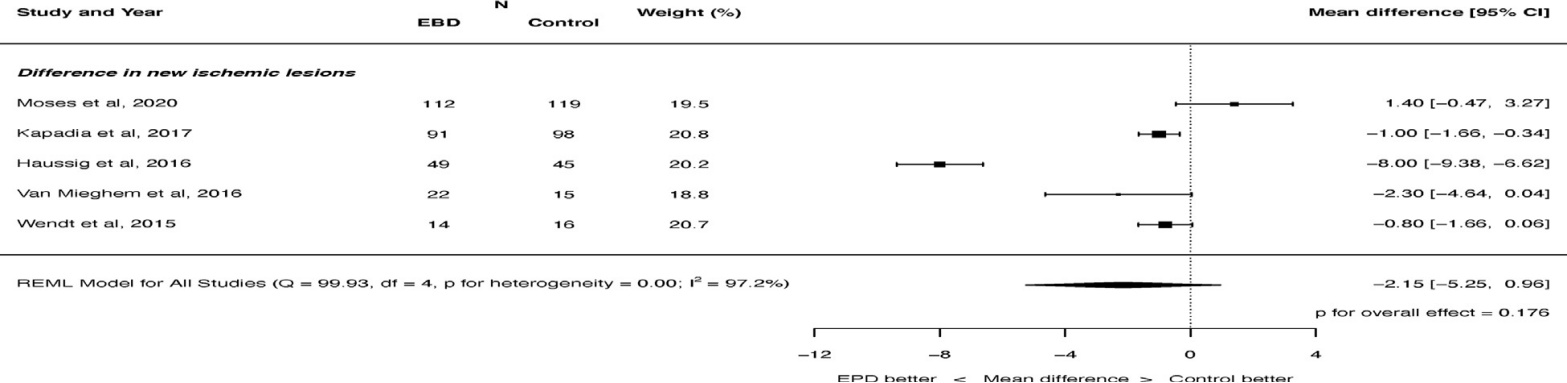
Effect of cerebral embolic protection on difference in new ischemic lesions. REML = restricted maximum likelihood.

There was no significant difference in total lesion volume on MRI with cerebral embolic protection: mean difference -74.94, 95% CI -174.31 to 24.4, p = 0.139 (Figure 4). There was significant heterogeneity (I^2^ = 95.8%). There was also not a significant difference in the number of new ischemic lesions on MRI: mean difference -2.15, 95% -5.25 to 0.96, p = 0.176 (Figure 5). There was significant heterogeneity (I^2^ = 97.2%). There was no difference in the number of patients seen to have new ischemic lesions on MRI: RR 0.99, 95% CI 0.94 to 1.05, p = 0.794. There was no heterogeneity (I^2^ = 0.0%).

All results were consistent when analyzed by fixed effect (see Supplementary Appendix). Neuroimaging end points were also analyzed using standardized mean difference, the results of which are shown in the Supplementary Appendix.

## Discussion

In this study, encompassing the totality of the randomized trial data for cerebral embolic protection during TAVI, we have shown that there is currently not a detectible statistically significant benefit of cerebral protection in terms of clinical stroke, and nor is there a statistically significant reduction in terms of total lesion volume or new cerebral lesions on MRI. The use of cerebral embolic protection appears to be safe, with no significant increase in any adverse event.

Despite significant advancements in TAVI technology and techniques, stroke rates have been largely static. The TVT registry has shown no significant decline for in-hospital strokes after TAVI between 2012 and 2019 with rates of roughly 2% per year. Stroke can have devastating clinical consequences as well as a significant economic impact, and patients consider stroke the most important clinical end point, even above death.^20^ There has thus been significant interest in strategies to reduce the risk of stroke after TAVI. To that end, cerebral embolic protection devices have been developed to mitigate stroke risk. Stroke is a relatively rare event, and the total number of patients randomized in cerebral protection trials to date is small; therefore, meta-analysis is appropriate to pool their results. In this analysis the risk of stroke was not significantly reduced by the use of cerebral embolic protection, although it must be noted that even this pooled analysis is underpowered to detect a significant effect on clinical stroke reduction.

In addition to preventing clinically-overt stroke, embolic protection may be postulated to prevent embolic debris to the brain which may have longer-term implications on neurocognitive function. The results of the present analysis do not demonstrate a significant reduction in total lesion volume or number of new lesions on MRI. This may be related to device factors, for example due to incomplete cerebral coverage during protection with filter-based devices. It is also conceivable that the act of manipulation of devices in the aortic arch itself can lead to debris travelling to the brain, thus obviating some benefit.

The results of this analysis are consistent with a recently presented analysis from the TVT registry.^21^ That observational study, using an instrumental variable analytic method, included over 120,000 patients and found no significant reduction in stroke with the use of cerebral embolic protection. This dataset also demonstrated that cerebral protection was used in 13% of patients in 2018 and 2019, with increasing utilization in each quarter as time went on. By the end of 2019, 8% of sites were using cerebral embolic protection in over 50% of cases despite there being no clinical trial evidence of a benefit on clinical outcomes.

There does not appear to be a clear penalty for using cerebral embolic protection, with no increased risk of major adverse events seen in this analysis such as vascular complications or acute kidney injury. However, there may be another more significant drawback to routine use of embolic protection; namely, hindrance of recruitment of patients into large-scale randomized clinical trials powered to detect an effect on clinical outcomes (NCT04149535). Rather than offering cerebral embolic protection as part of routine clinical care, operators should endeavor to randomize patients in order that a definitive answer can be provided on the role of cerebral embolic protection during TAVI.

We could only report the available data, and there are only six reported trials randomizing a total of 856 patients. The types of device are not uniform across trials, with some devices being filter-based to catch debris and some being deflectors. This is a potential source of heterogeneity, although there was zero or minimal heterogeneity observed for clinical outcomes. Larger scale trials are ongoing to help provide a more definitive answer on the role of cerebral embolic protection during TAVI. Clinicians should endeavor to recruit patients into these trials; until the results of the larger clinical trials is available, this analysis represents the best evidence available in the field.

This is a study-level analysis, so we could not delineate whether there are subgroups of patients who may benefit from embolic protection. It is possible that there are certain higher-risk features which may lead to benefit with cerebral protection, such as prior stroke, heavy aortic arch calcification or heavy valve calcification. An individual patient data meta-analysis may help to illuminate some of these points. We also did not perform subgroup analysis by type of embolic protection device or type of TAVI valve, due to the small number of trials and patients.

One of the included trials has not yet been published; if the results change significantly in the published manuscript, we will update this analysis accordingly.

Finally, our analysis only includes randomized trials which typically enroll a much narrower spectrum of patients than those seen in clinical practice. Although this can potentially limit the broader applicability of RCTs and meta-analysis of these trials, randomization is the only way to compare the efficacy and safety of competing therapies without the impact of bias from both measured and unmeasured confounding factors. Our analysis is also broadly consistent with a recent large-scale observational dataset using instrumental variable methodology to approximate randomization.

In conclusion, cerebral embolic protection during TAVI is safe but there is no evidence of a statistically significant benefit on clinical outcomes or neuroimaging parameters. The use of cerebral embolic protection during TAVI should be restricted to randomized clinical trials, or in selected high-risk cases where clinical judgement suggests a role.

## Disclosures

None.

## Declaration of Competing Interest

The authors declare that they have no known competing financial interests or personal relationships that could have appeared to influence the work reported in this paper.

## Appendix Supplementary materials

Image, application 1

## Footnotes

Supplementary material associated with this article can be found in the online version at https://doi.org/10.1016/j.amjcard.2021.01.023.
