# Supplementary material for: Meta-Analysis of Usefulness of Cerebral Embolic Protection During Transcatheter Aortic Valve Implantation
Source: Am J Cardiol. 2021 May 1;146:69–73. doi: 10.1016/j.amjcard.2021.01.023 (PMC8082278; doi:10.1016/j.amjcard.2021.01.023)
Supplement: Supplementary file 1 [file mmc1.docx]

**Supplementary Appendix**

**Table 1. Characteristics of Included Studies**

| **Author** | **Study acronym** | **Year** | **Region** | **N** | **Mean Age*** | **Follow up** | **Entry criteria** | **Device type** | **TAVR Type** | **Primary outcome§** |
| --- | --- | --- | --- | --- | --- | --- | --- | --- | --- | --- |
| Moses *et al.* | REFLECT II | 2020 | USA | 231 | 79.7  (±8) in embolic protection group  79.88 (±8) in control group | 30 days | Severe native aortic valve stenosis with planned transfemoral treatment with an FDA approved TAVR system  **Exclusion criteria:**  Prior AVR  Stroke/TIA < 6 months  Contraindication to antiplatelet or anticoagulation treatment  eGFR <30 ml/min  CT angiograms of the chest, abdomen, and pelvis were analyzed by the independent CT core lab and reviewed by a screening committee  Severe peripheral vascular disease (iliofemoral MLD <3.5mm)  Severely calcified or atheromatous aorta  Contraindication to MRI | TriGUARD 3 | Any approved valve (62.4% balloon-expandable)  100% transfemoral | Composite of all-cause mortality, stroke, life-threatening or disabling bleeding, stage 2/3 acute kidney injury, coronary artery obstruction requiring intervention, major vascular complication, and valve-related dysfunction requiring intervention (VARC 2 defined) |
| Kapadia *et al.* | SENTINEL | 2017 | USA and Germany | 363 | 83.4 (78.0-88.2) | 30 days | Severe  symptomatic aortic stenosis and planned TAVR who were at high surgical risk  **Key exclusion criteria:**  Contraindications for right radial or brachial artery  access and inability to undergo MRI brain evaluation  for any reason | Claret Sentinel | Any approved valve (70.2% balloon-expandable) | **Primary safety:**  Occurrence of major adverse cardiac and cerebrovascular  events (MACCE) at 30 days compared  with a historical performance goal  **Primary efficacy:**  Reduction in  median total new lesion volume in protected territories  between the device and control arms, as  assessed by diffusion-weighted MRI at 2 to 7 days after  TAVR |
| Haussig *et al.* | CLEAN-TAVI | 2016 | Germany | 100 | 80.0 (± 5.1) in embolic-protection group  79.1 (4.1) in the control group | 7 days | Symptomatic severe aortic stenosis  **Key exclusion criteria:**  Anatomy unsuitable for a safe TAVI, preexisting  permanent pacemaker, stroke within the last 12 months,  carotid artery stenosis of more than 70%, significant stenosis  of the right subclavian artery or the brachiocephalic trunk | Claret Montage | CoreValve | Numerical difference in new  positive postprocedure diffusion weighted MRI brain lesions at 2 days after TAVI in potentially protected  territories. |
| Van Mieghem *et al.* | MISTRAL-C | 2016 | Europe | 65 | 81 (78-85) | 30 days | Symptomatic severe aortic stenosis planned for transfemoral TAVR    **Key exclusion criteria:**  Presence of  a permanent pacemaker or automated internal cardiac defibrillator  (AICD) at baseline, a history of prior stroke with sequelae and  dementia. | Claret Sentinel | Any available valve (69% balloon-expandable) | New cerebral  lesions by diffusion weighted MRI five to seven days after TAVI |
| Wendt *et al.* | EMBOL-X | 2015 | Germany | 30 | 81.1 (± 5.0) in embolic protection group  82.1 (± 4.1) in control group | 7 days | Consecutive patients with severe aortic stenosis undergoing transaortic TAVR | EMBOL-X | Sapien-XT | Number and size of  new ischemic  cerebral  lesions within 7  days after TAVR |
| Lansky *et al.* | DEFLECT III | 2015 | Europe and Israel | 85 | 82.5 (± 6.5) in embolic protection group  82.3 (± 6.0) in control group | 30 days | Severe symptomatic aortic stenosis referred for TAVI  **Key exclusion criteria:**  Recent myocardial infarction, prior stroke, cardiogenic shock,  contraindications to antiplatelet or anticoagulant therapy, heavily calcified or severely atheromatous aortic arch or  aortic arch anatomy that could prevent positioning and stability of the  device, had contraindications to cerebral MRI | TriGuard HDH | Any available (63.5% balloon-expandable) | In-hospital MACCE |

*Mean age ± SD given for overall population if provided; otherwise given for each group; if mean age not available, then median with IQR given

.

(TAVR – transcatheter aortic valve replacement; MACCE - Major Adverse Cardiovascular and Cerebrovascular Events)

**Table 2. Risk of Bias Assessment**

| **Trial** | Random sequence generation | Allocation concealment | Blinding of participants & personnel | Blinding of outcome assessment | Incomplete outcome data | Selective reporting | **Overall Quality** |
| --- | --- | --- | --- | --- | --- | --- | --- |
| **Moses et al..** | Unclear | Unclear | Low risk | Low risk | High risk | Low risk | **Moderate** |
| **Kapadia et al.** | Low risk | Low risk | Low risk | Low risk | High risk | Low risk | **High**. |
| **Haussig et al..** | Low risk | Low risk | Low risk | Low risk | Low risk | Low risk | **High** |
| **Van Mieghem et al..** | Unclear | Unclear | Low risk | Low risk | Low risk | Low risk | **Moderate** |
| **Wendt et al.** | Unclear | Unclear | Low risk | Low risk | Low risk | Low risk | **Moderate** |
| **Lansky et al.** | Unclear | Low risk | Low risk | Low risk | Low risk | Low risk | **High** |

**Figure 1.** Source of included studies


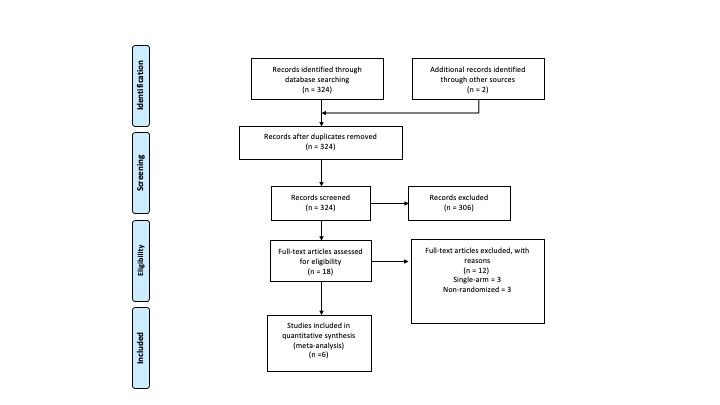


**Figure 2.** Effect of cerebral embolic protection on the risk of all stroke when analyzed by fixed effect

**Figure 3.** Effect of cerebral embolic protection on the risk of disabling stroke when analyzed by fixed effect.

**Figure 4.** Effect of cerebral embolic protection on the risk of non-disabling stroke when analyzed by fixed effect.

**Figure 5.** Effect of cerebral embolic protection on total lesion volume when analyzed by fixed effect.

**Figure 6.** Effect of cerebral embolic protection on difference in new ischemic lesions when analyzed by fixed effect.

**Figure 7.** Effect of cerebral embolic protection on risk of patient developing a new ischemic lesion.

**Figure 8.** Effect of cerebral embolic protection on difference in new ischemic lesions when analyzed by standardized mean difference
